# Stress-Responsive *cis*-Regulatory Elements Underline Podophyllotoxin Biosynthesis and Better Performance of *Sinopodophyllum hexandrum* Under Water Deficit Conditions

**DOI:** 10.3389/fpls.2021.751846

**Published:** 2022-01-04

**Authors:** Anita Kumari, Vivek Dogra, Rohit Joshi, Sanjay Kumar

**Affiliations:** ^1^Biotechnology Division, CSIR-Institute of Himalayan Bioresource Technology, Palampur, India; ^2^Academy of Scientific and Innovative Research (AcSIR), Ghaziabad, India

**Keywords:** adaptation, *cis*-element, podophyllotoxin (PTOX), secondary metabolism, water-deficit stress

## Abstract

*Sinopodophyllum hexandrum* is an endangered medicinal herb known for its bioactive lignan podophyllotoxin (PTOX), which is used for the preparation of anticancer drugs. In its natural habitat, *S. hexandrum* is exposed to a multitude of adversities, such as fluctuating temperatures, water deficit, and high UV radiations. Transcriptional regulation of genes, which is regulated by the condition-specific binding of transcriptional factors to precise motifs in the promoter region, underlines responses to an environmental cue. Therefore, analysis of promoter sequences could ascertain the spatio-temporal expression of genes and overall stress responses. Unavailability of genomic information does not permit such analysis in *S. hexandrum*, especially on regulation of PTOX pathway. Accordingly, this study describes isolation and *in silico* analysis of 5′-upstream regions of *ShPLR* (*PINORESINOL-LARICIRESINOL REDUCTASE*) and *ShSLD* (*SECOISOLARICIRESINOL DEHYDROGENASE*), the two key genes of the PTOX biosynthetic pathway. Data showed a range of motifs related to basal transcription, stress-responsive elements, such as those for drought, low temperature, and light, suggesting that the expression of these genes and resulting PTOX accumulation would be affected by, at least, these environmental cues. While the impact of temperature and light on PTOX accumulation is well studied, the effect of water deficit on the physiology of *S. hexandrum* and PTOX accumulation remains obscure. Given the presence of drought-responsive elements in the promoters of the key genes, the impact of water deficit on growth and development and PTOX accumulation was studied. The results showed decline in relative water content and net photosynthetic rate, and increase in relative electrolyte leakage with stress progression. Plants under stress exhibited a reduction in transpiration rate and chlorophyll content, with a gradual increase in osmoprotectant content. Besides, stressed plants showed an increase in the expression of genes involved in the phenylpropanoid pathway and PTOX biosynthesis, and an increase in PTOX accumulation. Upon re-watering, non-irrigated plants showed a significant improvement in biochemical and physiological parameters. Summarily, our results demonstrated the importance of osmoprotectants during water deficit and the revival capacity of the species from water deficit, wherein PTOX synthesis was also modulated. Moreover, isolated promoter sequences could be employed in genetic transformation to mediate the expression of stress-induced genes in other plant systems.

## Introduction

*Sinopodophyllum hexandrum* (Royle) is a well-known endangered medicinal herb that contains a medicinally important lignan, podophyllotoxin (PTOX), in its underground parts, rhizomes, and roots. PTOX is used as a precursor for anticancer drugs, such as etoposide and teniposide (Wang et al., 2013; Rather and Amin, 2016; Kumari et al., 2017). As a bioactive compound, PTOX also provides the substrate for the synthesis of botanical pesticides (Lv et al., 2020). *S. hexandrum* can propagate through seeds and rhizomes; however, due to the poor and erratic seed germination (Kharkwal et al., 2008; Sreenivasulu et al., 2009; Dogra et al., 2013), the rhizome is the prime method of reproduction. Because of the presence of PTOX in underground parts, the whole plant is uprooted, thus, resulting in the rapid extinction of the species from its natural habitat. As an outcome, *S. hexandrum* is listed as an endangered species in the Indian Himalayas (Kala, 2000; Shah, 2006).

Besides human interference, environmental adversities in the natural habitat also influence the survival and propagation of several high-altitude plant species such as *S. hexandrum*. These adversities include fluctuating temperature (warmer days and cooler nights), high-intensity radiations such as UV-B, low partial pressure of gases, and inadequate availability of water, which at times can create drought-like conditions (Tewari et al., 2017). Despite such unfavorable conditions, *S. hexandrum* can sustain and complete its life cycle (Kumari et al., 2014). Deciphering the mechanism(s) by which *S. hexandrum* deals with these cues is essential to understand the adaptability of this high-altitude extremophile.

Although the complete pathway of PTOX biosynthesis remains to be elucidated, a putative pathway has been derived from studies on *S. hexandrum*, *Anthriscus*, *Linum*, and *Dysosma* (Kumari et al., 2017). The suggested pathway involves a general phenylpropanoid pathway that diverges after the formation of feruloyl-CoA (Supplementary Figure 1). Briefly, the phenylpropanoid pathway initiates with the conversion of phenylalanine to cinnamic acid by PHENYLALANINE AMMONIA LYASE (PAL). Cinnamic acid is converted to *p*-coumarate by CINNAMATE-4-HYDROXYLASE (C4H), which is further converted to *p*-coumaroyl CoA by 4-COUMAROYL CoA LIGASE (4CL) (Vogt, 2010). *p*-Coumaroyl CoA is converted into *p*-Coumaroyl shikimate by HYDROXYCINNAMOYL COA:SHIKIMATE HYDROXYCINNAMOYL TRANSFERASE (HCT). COUMARATE 3-HYDROXYLASE (C3H) catalyzes the hydroxylation of *p*-Coumaroyl shikimate to caffeoyl shikimate that, in turn, is converted to caffeoyl-CoA by HCT. Caffeoyl-CoA is methylated to feruloyl-CoA by CAFFEOYL-CoA *O*-METHYLTRANSFERASE (CCoAOMT). Caffeoyl-CoA can also be converted to caffeic acid by 4-COUMAROYL CoA LIGASE (4CL). Caffeic acid is methylated to ferulic acid by CAFFEIC ACID 3-*O*-METHYLTRANSFERASE (COMT). Ferulic acid then acts as a substrate for the synthesis of sinapic acid and lignin. In addition, ferulic acid can also be converted to feruloyl-CoA by 4CL. The conversion of feruloyl-CoA to coniferaldehyde is catalyzed by CINNAMOYL-CoA REDUCTASE (CCR). CINNAMYL ALCOHOL DEHYDROGENASE (CAD) coverts coniferaldehyde to coniferyl alcohol, which then undergoes a dimerization reaction by DIRIGENT PROTEIN OXIDASE (DPO/DIR) to form pinoresinol. Pinoresinol acts as a branching point for the generation of a diversity of lignans. For PTOX biosynthesis, in an enantiospecific manner, PINORESINOL-LARICIRESINOL REDUCTASE (PLR) reduces pinoresinol in two steps to secoisolariciresinol, which is dehydrogenated to matairesinol by SECOISOLARICIRESINOL DEHYDROGENASE (SLD/SDH). Matairesinol is then converted to deoxypodophyllotoxin in five steps catalyzed by unknown enzymes. Deoxypodophyllotoxin is the precursor of PTOX and is proposed to be converted by DEOXYPODOPHYLLOTOXIN 7-HYDROXYLASE (DOP7H) (Supplementary Figure 1).

The sessile nature of plants necessitates rapid alteration of physiology and metabolic activities to endure adverse conditions in their habitat. Such alteration is governed by transcriptional regulation, which is mainly achieved by the binding of specific transcriptional factors to respective promoters. Transcriptional reprogramming then activates an array of signal transduction processes required to limit damage (Jin et al., 2019). Promoters are generally 5′-upstream DNA sequences governing the transcription of genes and are crucial targets to understand the molecular mechanism of various pathways. The binding between transcription factors (TFs) and *cis*-regulatory DNA sequences in the 5′-upstream of genes either activates or represses gene expression (Hernandez-Garcia and Finer, 2014; Biłas et al., 2016). In general, genes with related expression patterns contain common regulatory motifs in their promoter region and are likely to be regulated by common TFs (Klok et al., 2002). Characterization of genes and promoters of the PTOX biosynthesis pathway would answer the long-standing questions on: (i) response of PTOX biosynthesis to environmental variation(s) and (ii) regulation of the PTOX biosynthesis pathway. However, lack of genomic information in *S. hexandrum* limits answering these questions. As a first step, the issue can be dealt by cloning and characterization of full-length genes and cognate promoter sequences of the PTOX pathway (Kumari et al., 2014).

Given the natural habitat of *S. hexandrum* in the Himalayas at elevations ranging from 2,400 to 4,500 m above sea level (Kumari et al., 2014; Liu et al., 2014), fluctuating water availability and increased water deficit resulting from freezing and higher light intensity are anticipated to induce drought-like conditions. In addition, the changing climate leading to an increase in carbon dioxide levels and temperature (NOAA, 2020a,b) is also expected to increase drought or water deficit frequency in high altitude ecosystems (Cotado et al., 2020). Among various cues encountered in the natural habitat, the impact of temperature and light on growth and PTOX biosynthesis has been studied in *S. hexandrum* (Singh and Purohit, 1998; Kumari et al., 2014; Li et al., 2020; Lv et al., 2020). A previous study reported the impact of altitudinal gradient in Zanskar valley, a semi-cold desert region in India with limited water availability, on lignan content in *S. hexandrum* (Kitchlu et al., 2011), wherein the highest content of PTOX was recorded in plants collected from Tangoli (3,000 m asl) and Padam (3,800 m asl) as compared to those collected from Panikhar (2,800 m asl). The study discussed the positive impact of stress on the enhancement of PTOX content. However, this was a preliminary report and warranted an in-depth analysis.

In general, water deficit leads to both morphological and molecular alterations that are manifested in all phases of plant growth and development (Xu C. et al., 2019). Reduced water availability results in loss of turgor and impairment of growth, primarily because of reduction in gas exchange, photosynthetic efficiency, and nutrient uptake, and it assimilates partitioning (Farooq et al., 2009). The response towards water deficit is determined essentially by the severity and duration of water unavailability (Osmolovskaya et al., 2018). The adaptation of plants to endure water deficit involves the instigation of various physiological, biochemical, and molecular responses. These changes include osmotic adjustments, accumulation of antioxidants and secondary metabolites, and reduced assembly of the photosynthetic complex to prevent the generation of excess reactive oxygen species (ROS) (Laxa et al., 2019). Most of these changes are directed by the transcriptional expression of cognate genes that can be activated either by stress directly or *via* secondary signaling molecules (Janiak et al., 2016). Water deficit was also shown to reconfigure the phytoconstituent compositions in several plant species cultivated under semi-arid conditions (Farahani et al., 2009). Since water deficit profoundly affects physiology and metabolism (both primary and secondary) in plants (Osmolovskaya et al., 2018), it would be interesting to study how it affects the biosynthesis of PTOX in *S. hexandrum*.

With this background, this study describes isolation and analysis of promoter region of two genes of the PTOX biosynthesis pathway, namely, *ShPLR* and *ShSLD*. The upstream regions of both genes contain specific abiotic stress- and drought-responsive elements in addition to low temperature and high light-responsive elements, which suggested transcriptional reprogramming of these genes under respective environmental cues. Interestingly, *S. hexandrum* plants exhibited better water retention and accumulated osmoprotectants in response to drought, which perhaps protected the plants. Also, the genes involved in the phenylpropanoid and PTOX biosynthesis pathways were upregulated, leading to increased accumulation of PTOX under reduced water availability.

## Materials and Methods

### Plant Material and Growth Conditions

Plants of *S. hexandrum*, collected from the natural habitat (Parashar lake, Mandi; 2,730 m asl; 30°12′ N 77°47′ E, India), and maintained at CSIR-Institute of Himalayan Bioresource Technology, Palampur (1,300 m asl; 32°06′ N, 76°33′ E, India) were used for the experimental purpose. To avoid the impact of age/rhizome size, morphologically similar plants having comparable leaf and root size without any rhizome formation were selected for this study. The Biological Diversity Act 2002 of India permits bonafide Indians to collect plant samples from native habitats for scientific investigations (Venkataraman, 2009).

The plants were grown in potting mix (constituted of soil, sand, and farmyard manure in a ratio of 2:1:1) under a long day (16 h) photoperiod in a plant growth chamber (Percival Scientific, Perry, IA, United States) set at 25 ± 2°C with (photon flux density of 200 μmol m^–2^s^–1^ and relative humidity of 70 ± 10%), as described earlier (Kumari et al., 2014).

### Isolation of 5′-Upstream Sequences and *in silico* Analysis

Two genes, namely *ShPLR* and *ShSLD*, involved in dedicated steps of PTOX biosynthesis pathway in *S. hexandrum* were selected for cloning of 5′-upstream sequences. Full-length coding sequences (CDS) of these genes were submitted at NCBI by our group. The accession numbers were EU240218 and GU324975 for *ShPLR* and *ShSLD*, respectively. The 5′-upstream sequences were cloned using Genome-Walker™ kit (Clontech, Mountain View, CA, United States). Briefly, genomic DNA was extracted from the leaf tissue using cetyltrimethylammonium bromide (CTAB) method (Doyle and Doyle, 1987). Four DNA libraries (DLs 1-4) were prepared by digesting the DNA separately with blunt-end digestive enzymes, namely, *Dra*I (for DL1), *Eco*RV (for DL2), *Puv*II (for DL3), and *Stu*I (for DL4). The digested products were purified and ligated to the genome walker adaptors. PCR-based DNA walking was performed with gene-specific primers (GSPs; designed from a region close to the 5′-end of the target genes; Supplementary Table 1) and adaptor primers AP1 and AP2 (provided in the GenomeWalker kit) using four libraries as templates. The PCR products were electrophoresed, gel-eluted, and ligated into a pGEM-T-Easy (Promega Corporation, Fitchburg, WI, United States) vector system. The ligated products were transformed into *Escherichia coli* DH5α competent cells, and the positive clones were screened by blue/white selection and colony PCR. Plasmids were isolated from the positive colonies and sequenced using a BigDye™ Terminator Cycle Sequencing kit (Applied Biosystems, Beverly, MA, United States) with an automated DNA sequencer, ABI 3130xl Genetic Analyzer (Applied Biosystems, Beverly, MA, United States). The primer sequences used for the cloning upstream sequences are listed in Supplementary Table 1. The upstream sequences were aligned using the BLASTN tool. *In silico* analyses of the retrieved promoter sequences were performed using online tools, PLACE^1^ and PlantCARE^2^ to identify putative *cis*-regulatory elements.

### Water Deficit Treatment

The plants in the plant growth chamber were exposed to progressive water deficit by withholding water as follows: one set of plants watered every alternate day with 150 ml of water is considered as irrigated. The other set was subjected to water deficit by withholding water for 5, 10, and 15 days (until the plants showed wilting and chlorosis) and was considered as non-irrigated. After 15 days of withholding water, the non-irrigated plants were re-watered every alternate day with 150 ml of water for additional 15 days until the plants exhibited a phenotype comparable to that of the irrigated ones. Leaf and root tissues were collected from the irrigated and non-irrigated plants for further analyses. All the experiments were performed using three independent biological replicates.

### Measurement of Soil Moisture Content, Relative Water Content, and Net Photosynthetic Rate

Soil moisture content (SMC) was measured using WET Sensor Moisture Meter (Delta-T Devices Ltd., Cambridge, United Kingdom) according to the manufacturer’s instructions. Relative water content (RWC) of the leaves was measured as described by Barrs and Weatherley (1962). Briefly, three leaf disks (10 mm diameter, average weight 45 ± 5 mg) were cut using a cork borer. Fresh weight (FW) was recorded, and the tissues were rehydrated with de-ionized (DI) water at 4°C for 24 h to record the turgid weight (TW). Rehydrated tissues were oven-dried at 70°C for 48 h, and the dry weight (DW) of the tissues was recorded. RWC was determined using the following formula: RWC (%) = [(FW – DW)/(TW – DW)] × 100.

A portable gas exchange measuring system, LI 6400 (IRGA; LI-COR Biosciences, Lincoln, United States), was used to measure net photosynthetic rate (*P*_*N*_). The measurements were made between 9 AM and 12 noon using 400 μmol mol^–1^ of CO_2_ and a photosynthetic photon flux density of 1,000 μmol m^–2^ s^–1.^ Data on fully expanded leaves were recorded in three separate biological replicates.

### Determination of Relative Electrolyte Leakage and Chlorophyll Content

Relative electrolyte leakage (REL) was determined in the leaf and root tissues according to the method described by Blum and Ebercon (1981), with slight modifications. Briefly, leaf disks (three in number, average weight 45 ± 5 mg/disk; cut using a cork borer of 10 mm diameter) were collected from the irrigated and non-irrigated plants and incubated for 24 h at 25°C in a glass vial containing 12 ml of deionized (DI) water. Electrical conductivity (EC_1_) was measured using a CyberScan PC 510 (Eutech Instruments Ltd., Singapore) conductivity meter. The tissues in the DI water were autoclaved, cooled, and final electrical conductivity (EC_2_) was measured. REL was calculated using the following formula: REL (%) = (EC_1_/EC_2_) × 100.

Total chlorophyll content was calculated using the method described by Arnon (1949). Briefly, 100 mg of leaf tissue was homogenized in liquid nitrogen followed by extraction with 10 ml of 80% acetone. The homogenate was centrifuged at 3,000 rpm for 10 min at 4°C in 3K30 refrigerated centrifuge (Sigma, Germany) to collect the supernatant. The absorbance of the supernatant was recorded at 645 and 663 nm using a SPECORD 200 (Analytik Jena, Jena, Germany) UV/visible spectrophotometer against a blank solvent (80% acetone). Chlorophyll content was calculated using the equation as follows: Chl (mg/l) = 20.2 Abs_645_ + 8.02 Abs_663_ and normalized on per g of fresh weight.

### Measurement of Proline Content

Proline content was analyzed using the method of Bates et al. (1973), which is based on the formation of a toluene-soluble, brick-red-colored formazan by proline with ninhydrin in an acidic medium. Briefly, 300 mg of leaf tissue was ground to a fine powder in liquid nitrogen followed by extraction with 2 ml of 3% sulfosalicylic acid. The supernatant was collected through centrifugation at 13,000 rpm for 10 min at room temperature. The supernatant (600 μl) was collected and reacted with glacial acetic acid (600 μl) and an acidic ninhydrin reagent (600 μl, prepared by warming 1.25 g ninhydrin in 30 ml glacial acetic acid and 20 ml 6 M phosphoric acid) for 1 h at 100°C in a dry bath with moderate shaking. The reaction was terminated by cooling the tube on an ice bath; 1 ml of toluene was added to the reaction mixture, which was vortexed briefly to mix. Chromophore-containing toluene was extracted, and absorbance was recorded at 520 nm using a SPECORD 200 (Analytik Jena, Jena, Germany) UV/visible spectrophotometer and calculated using the standard curve of proline prepared using L-proline (cat no. P0380; Sigma-Aldrich, St. Louis, MO, United States).

### Quantification of Soluble Sugars and Starch

For analysis of extractable soluble sugar, 100 mg of tissue was ground in liquid nitrogen followed by three times extraction with 5 ml of 80% ethanol (Chow and Landhäusser, 2004). After each extraction, tubes were centrifuged at 2,500 rpm for 10 min at 4°C; the supernatant was collected, pooled, and freeze-dried in a lyophilizer (Heto Maxi Dry Lyo, Germany). The residue was dissolved in 20 ml of 100 mM NaOH, and 10 ml of aliquot was used for the quantification of sugars. For starch analysis, the leftover pellet, after soluble sugar extraction, was treated with 50 ml of 35% perchloric acid at 90°C for 1 h. This was followed by filtration using a Whatman filter, and 10 ml aliquot was also used for starch determination. Soluble sugars (inositol, fructose, glucose, and sucrose) and starch content were quantified by ion chromatography (IC) using a Metrohm IC (Metrohm, Herisau, Switzerland) system equipped with an autosampler (45 IC Autosampler plus 1) and a pulsed amperometric detector (PAD) (945 Professional IC Vario). IC separation was performed on a Hamilton RCX-30 column (7 μm, 4.6 mm × 250 mm) using an isocratic mobile phase consisting of 100 mM NaOH. The injection volume was 20 μl, and an isocratic elution was carried out at a flow rate of 1 ml/min and 10.6 MPa of pressure. Analysis settings for sugars are provided by the company on its website^3^. Quantification was performed using external sugar standards.

### Extraction and Estimation of Podophyllotoxin Content

Podophyllotoxin (PTOX) was extracted and estimated in root tissues as described previously (Kumari et al., 2014); PTOX content was not detectable in leaf tissue. Briefly, 100 mg root tissue was ground to a fine powder in liquid nitrogen, followed by intermittent grinding in 1 ml of 70% methanol. The resulting extract was transferred to a centrifuge tube, vortexed for 5 min, sonicated (Ultrasonic Cleaner, MC-109-MP; Oscar Ultrasonics Pvt. Ltd., Mumbai, India) for 10 min at 25°C, and centrifuged at 14,000 rpm for 5 min. The supernatant was collected and filtered through 0.22-μm filter (Millipore, Burlington, MA, United States). PTOX content was estimated using Acquity UPLC (Waters, Millford, MA, United States) equipped with a bridged ethyl hybrid workflow shield C18 (1.7-μm particles, 2.1 mm × 100 mm) analytical column (Waters Corp., Manchester, United Kingdom). Injection volume was 5 μl, and the mobile phase consisted of methanol:water (60:40); isocratic elution was carried out at a flow rate of 0.25 ml min^–1^. PTOX was monitored at 240 nm and quantified using standard PTOX (Sigma, United States). Three independent biological replicates were used for PTOX estimation.

### Expression Analysis of Genes Involved in Podophyllotoxin Biosynthetic Pathway

Total RNA was isolated from the root tissues, as described earlier (Ghawana et al., 2011). RNA was reverse-transcribed using Superscript III (Invitrogen, Waltham, MA, United States), and the resulting cDNA was used for quantitative reverse transcription-PCR (qRT-PCR). Reactions were performed with three independent biological replicates using *S. hexandrum ACTIN* as an internal standard. Relative expression profile was generated using the Relative Expression Software Tool REST-2009 (Pfaffl, 2012). The primers used for qRT-PCR are listed in Supplementary Table 2.

### Statistical Analysis

For each experiment, three independent biological replicates were used. The statistical significance of the data was established by one-way ANOVA with Duncan’s Multiple Range Test (*P* < 0.05) using Statistica version 7.0 (StatSoft Inc., United States).

## Results and Discussion

### Isolation and Identification of *cis*-Regulatory Elements in *ShPLR* and *ShSLD*

**FIGURE 1:**
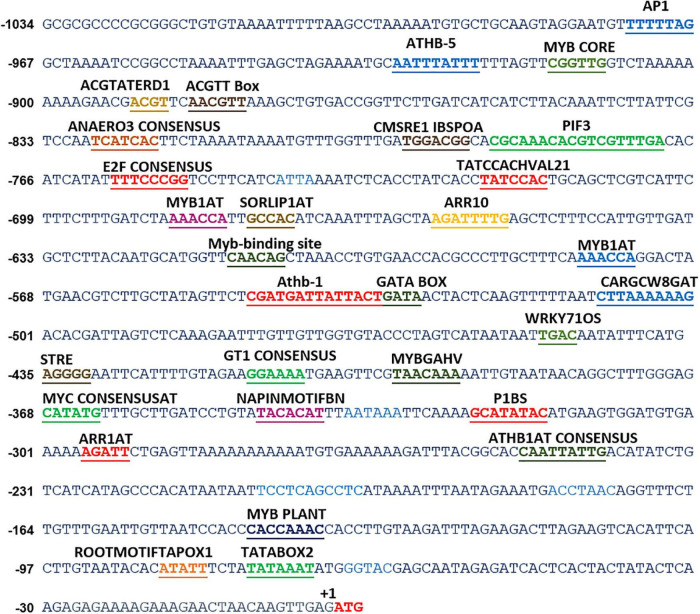
*In silico* analyses of various *cis*-acting elements in *ShPLR*. The analysis indicated the presence of various stress-responsive elements, such as those responding to light, temperature, and dehydration cues. Putative *cis*-acting regulatory elements are underlined. +1 indicates the translation start site.

**FIGURE 2:**
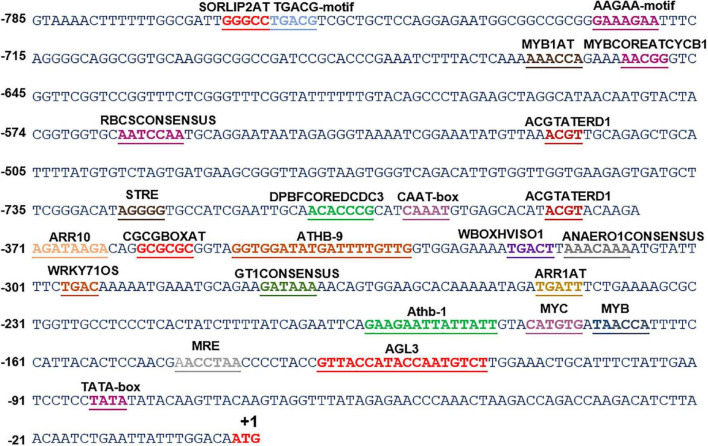
*In silico* analysis of various *cis*-acting elements in *ShSLD*. The analysis indicated the presence of various stress-responsive elements, such as those responding to light, temperature, and dehydration cues. Putative *cis*-acting regulatory elements are named and underlined. +1 indicates the translation start site.

Deoxyribonucleic acid (DNA) sequences, generally the 5′-upstream of a gene, contain regulatory regions, namely, enhancers, silencers, and *cis*-acting motifs for the regulation of gene expression. Among these, *cis*-acting motifs provide sites for the binding of specific transcription factors, which can either enhance or repress the transcription. Therefore, analysis and functional validation of these *cis*-acting motifs in the promoter region holds high significance in understanding the transcriptional regulation of genes under different cues. For instance, the identification and characterization of stress-inducible promoters and *cis*-acting elements could be exploited for genetic transformation to generate stress-tolerant transgenic plants (Hernandez-Garcia and Finer, 2014). Lack of genomic information in *S. hexandrum* limits molecular exploration, such as the understanding of the molecular regulation of PTOX biosynthetic pathway. To deal with this limitation, we cloned 5′-upstream sequences of two genes for which the full-length CDSs were deduced earlier in our laboratory (*ShPLR*; EU240218, and *ShSLD*; GU324975). The Genome Walk PCR, using the four libraries (DLs 1-4), obtained multiple products of various sizes in both primary and secondary PCRs (Supplementary Figure 2). Among these products, fragments of ∼1 kb in DL1 in *ShPLR* and ∼700 bp in DL3 in *ShSLD* could be successfully cloned and sequenced (Supplementary Figure 2). The Genome Walking approach, thus, obtained a 1,034-bp 5′-upstream region of *ShPLR* (Figure 1 and Supplementary Figure 2), and a 785-bp region for *ShSLD* (Figure 2 and Supplementary Figure 2). Our previous experience in characterizing promoters of similar lengths in other plant species suggested that the present length of promoters was large enough to provide useful regulatory sequences controlling gene expression (Kawoosa et al., 2010, 2014; Bhuria et al., 2016). The promoter sequences were submitted to NCBI with accession numbers such as OK574340 for *ShPLR* and OK574341 for *ShSLD*.

*In silico* analysis of these putative promoter sequences revealed that the core promoter regions contain conserved TATA and CAAT boxes. In addition to core motifs, several regulatory motifs known to bind to light-, low- temperature-, drought- and hormone-responsive elements were identified in the upstream region of both genes. These motifs include those related to low temperature, light, ABA, drought/dehydration, gibberellins, cytokinin, auxin, signal transduction, leaf/plastid specificity, cell division, and biotic factors (Figures 1, 2 and Tables 1, 2).

**TABLE 1:** Selected *cis*-acting motifs and their location in the 5′-upstream sequence of *ShPLR*.

*Cis*-acting elements	Position	Sequence	Function
TATABOX2	−69	TATAAAT	Core promoter element
ROOTMOTIFTAPOX1	−80	ATATT	Required for organ specificity
MYBPLANT	−135	CACCAAAC	**Involved in regulating phenylpropanoid and lignin biosynthesis**
ATHB1ATCONSENSUS	−240	CAATTATTG	Recognition sequence of Arabidopsis Athb-1 protein
ARR1AT	−289	AGATT	ARR1 is a cytokinin response regulator
P1BS	−313	GCATATAC	Involved in phosphate starvation signaling
NAPINMOTIFBN	−336	TACACAT	Sequence found in 5′ upstream region (−6, −95, −188) of napin (2Salbumin) gene in *Brassica napus*
MYCCONSENSUSAT	−359	CATATG	Regulates the transcription of CBF/DREB1 genes
MYBGAHV	−389	TAACAAA	Transcriptional factor required for transcriptional activation of the high-pI alpha-amylase
GT1CONSENSUS	−405	GGAAAA	**Involved in light response**
STRE	−427	AGGGG	**Stress responsive element**
WRKY71OS	−443	TGAC	Binding site of rice WRKY71, a transcriptional repressor of the gibberellin signaling pathway
CARGCW8GAT	−498	CTTAAAAAAG	Binding site for AGL15 (AGAMOUS-like 15)
GATABOX	−526	GATA	Required for light-regulated, and tissue-specific expression
ATHB-1	−530	CGATGATTATTACT	Recognition sequence of Arabidopsis Athb-1 protein
MYB1AT	−571	AAACCA	**MYB recognition site found in the promoters of the dehydration-responsive gene**
Myb-binding site	−607	CAACAG	**Involved in drought inducibility**
ARR10	−651	AGATTTTG	Involved in His-to-Asp phosphorelay signal transduction system
SORLIP1AT	−673	GCCAC	**Involved in light response**
MYB1AT	−680	AAACCA	**MYB recognition site found in the promoters of the dehydration-responsive gene**
TATCCACHVAL21	−714	TATCCAC	Involved in full gibberellin response
E2FCONSENSUS	−751	TTTCCCGG	Involved in controlling in cell cycle
PIF3	−769	CGCAAACACGTCGTTTGA	**Involved in light response**
CMSRE1IBSPOA	−789	TGGACGG	CMSRE-1 (Carbohydrate Metabolite Signal Responsive Element 1)
ANAERO3CONSENSUS	−821	TCATCAC	Motifs in promoters of anaerobically induced genes
ACGTTBOX	−879	AACGTT	**Stress responsive element**
ACGTATERD1	−887	ACGT	**Required for etiolation-induced expression of erd1 (early responsive to dehydration)**
MYBCORE	−909	CGGTTG	**Involved in regulation of genes that are responsive to water stress; High salinity, ABA, heat, cold, and dehydration responsive**
ATHB-5	−922	AATTTATTT	Potential regulator of abscisic acid responsiveness
AP1	−967	TTTTTAG	Promotes early floral meristem identity

*Possible functions of the identified regulatory motifs are also mentioned. Motifs implicated in light, temperature, and dehydration stress are indicated in bold.*

**TABLE 2:** Selected *cis*-acting motifs and their location in the 5′-upstream sequence of *ShSLD*.

*Cis*-acting elements	Position	Sequence	Function
TATA-box	−81	TATA	Core promoter element
AGL3	−113	GTTACCATACCAATGTCT	Involved in determination of flower meristem identity
MRE	−139	AACCTAA	**MYB binding site involved in light response**
MYB	−166	TAACCA	**MYB recognition site found in the promoters of the dehydration-responsive genes**
MYC	−173	CATGTG	**MYC recognition sequence necessary for expression of erd1 (early responsive to dehydration)**
ATHB-1	−182	GAAGAATTATTATT	Recognition sequence of Arabidopsis Athb-1 protein
ARR1AT	−243	TGATT	ARR1 is a cytokinin response regulator
GT1CONSENSUS	−271	GATAAA	**Involved in light response**
WRKY71OS	−294	TGAC	For gibberellin and pathogenesis
ANAERO1CONSENSUS	−308	AAACAAA	Motifs in promoters of anaerobically induced genes
WBOXHVISO1	−316	TGACT	Involved in sugar signaling
ATHB-9	−331	GGTGGATATGATTTTGTTG	Determination of adaxial-abaxial polarity
CGCGBOXAT	−354	GCGCGC	Involved in multiple signaling pathways in plants
ARR10	−363	AGATAAGA	Involved in His-to-Asp phosphorelay signal transduction system.
ACGTATERD1	−377	ACGT	**Required for etiolation-induced expression of erd1 (early responsive to dehydration)**
CAAT-box	−391	CAAAT	Common *cis*-element in promoter and enhancer regions involved in tissue-specific promoter activity
DPBFCOREDCDC3	−399	ACACCCG	ABA-responsive and embryo-specification elements
STRE	−421	AGGGG	**Stress-responsive element**
RBCSCONSENSUS	−559	AATCCAA	**Influences the level of gene expression and involvement in light-regulated gene expression**
MYBCOREATCYCB1	−648	AACGG	Involved in cell cycle phase-independent activation of Arath; CycB1; 1 transcription and identification of putative regulatory proteins
ARE	−657	AAACCA	*Cis*-acting regulatory element essential for anaerobic induction
AAGAA-motif	−719	GAAAGAA	Unknown
TGACG-motif	−755	TGACG	*Cis*-acting regulatory element involved in the MeJA-responsiveness
SORLIP2AT	−760	GGGCC	**Involved in light response**

*Possible functions of the identified regulatory motifs are also mentioned. Motifs implicated in light, temperature, and dehydration stress are indicated in bold.*

Upstream region of *ShPLR* contains several drought-responsive *cis*-acting elements such as MYB transcription factor-binding sites, also called MYB-recognizing elements (MREs), which include MYBPLANT, MYB4, MYB1AT, MYBCORE, ACGTATERD1 (Figure 1 and Table 1). MREs regulate phenylpropanoid metabolism in plants, where binding of MYB transcription factors drives the expression of associated genes (Liu et al., 2015). With drought or ABA stress, MYBs bind key MREs to drive the expression of phenylpropanoid biosynthetic and stress-responsive genes (Xu Z. et al., 2019). Among the MYB binding sites, the MYBPLANT motif is reported to be involved in regulating phenylpropanoid and lignin biosynthesis in transgenic tobacco (Tamagnone et al., 1998), whereas MYBCORE, MYCCONSENSUSAT, MYB1AT, and ACGTATERD1 are recognized as dehydration-responsive elements (Abe et al., 2003). Similar to that of *ShPLR*, the upstream region of *ShSLD* also contains drought-responsive *cis*-acting elements, such as MRE, MYB, ACGTATERD1, and MYBCOREATCYCB1 (Figure 2 and Table 2). The MYC-like sequence (CATGTG) is known to be crucial for dehydration-induced expression of *Early response to Dehydration 1* (*ERD1*) (Simpson et al., 2003). The ARR1 and ARR10 elements are also found in the upstream region of *ShSLD*. These are important signaling components involved in abiotic stress response (Nguyen et al., 2016). Light responsive elements like SORLIP2AT and GT1CONSENSUS, and motifs such as ARE and ANAERO1CONSENSUS, were also found in the 5′-upstream region of *ShSLD*. Other *cis*-elements, such as CGCGBOXAT, a Ca^2+^/calmodulin regulatory element, ACGTATERD1, a specific drought-responsive element (Simpson et al., 2003), and DPBFCOREDCDC3, an ABA-responsive element, were also present.

Collectively, the upstream region of both *ShPLR* and *ShSLD* genes comprises several *cis*-regulatory elements responding to light, low temperature, and dehydration stress. Interestingly, both of the genes contain *cis*-elements that are recognized by MYB and MYC family transcription factors, indicating their involvement in stress responses, such as drought stress (Singh and Laxmi, 2015). The *in silico* analysis reinforced that *S. hexandrum* encounters various environmental variations, such as drought or drought-like water deficit conditions. It has been reported that, like other high-altitude plants, *S. hexandrum* also experiences incidence of inadequate water availability and drought-like conditions (Kitchlu et al., 2011). However, the impact of such stress conditions on the physiology and PTOX biosynthesis remains uninvestigated. Therefore, to further understand the possible role of drought-responsive elements in the promoters of PTOX biosynthetic pathway genes, the consequences of water deficit and subsequent rehydration on the physiological and molecular responses of *S. hexandrum* were studied.

### Reduction in Soil Moisture Content Affected Plant Phenotype, Relative Water Content, Photosynthetic Rate, and Relative Electrolyte Leakage in *S. hexandrum*

Drought or water deficit is a global stress that rigorously impacts plant productivity. While enormous literature exists on the impact of water deficit on crops and model plants, only a few studies have been carried out on high-altitude extremophile plants. Given the economic and medicinal importance of *S. hexandrum*, this study was performed to assess the impact of water deficit, which is prevalent at high altitudes.

**FIGURE 3:**
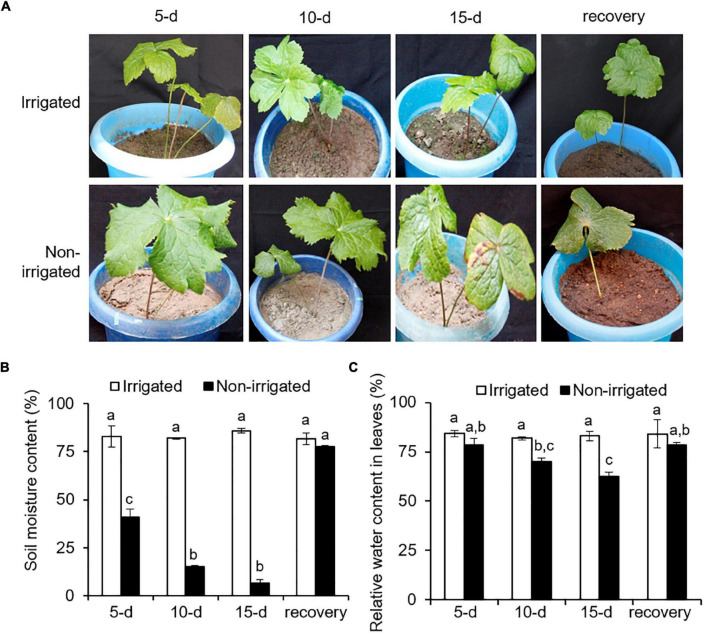
*Sinopodophyllum hexandrum* plants subjected to water deprivation showed a water deficit phenotype. **(A)** Plant phenotype. **(B)** Soil moisture content. **(C)** Relative water content in leaves. Watering of the plants was stopped, and plant phenotype and soil moisture content were recorded for 15 days with a 5-day interval, followed by re-watering for 15 days (recovery). Plants irrigated frequently served as control. In panels **(B,C)**, data values show mean ± SE of three independent biological replicates. Lowercase letters represent statistical significance of differences between the mean values (*P* < 0.05, Duncan’s multiple comparison test).

The plants were exposed to drought-like conditions by withholding water. The non-irrigated plants showed a drought-like phenotype, where wilting and chlorosis of the leaves were observed (Figure 3A and Supplementary Figure 3). During the experiment, the non-irrigated plants showed a decline in SMC as compared with the irrigated plants. The SMC of the irrigated plants was 83.1 ± 1.9%, whereas the non-irrigated plants showed a significant and gradual decrease in SMC by 40.8 ± 7.4% on day 5, 15 ± 1.6% on day 10, and 6.6 ± 3.4% on day 15 of water withholding (Figure 3B). Re-watering recovered the SMC to 77 ± 1.09%, which was similar to that in irrigated plants (Figure 3B). Non-availability of water and reduction of SMC influence plant water status (Furlan et al., 2012). To understand the impact of reduced SMC on plants *per se*, RWC in the plants was measured. On day 0, RWC in the leaf tissue was 82.8 ± 1.7%. With the onset of water deprivation, the RWC of the non-irrigated plants decreased and showed a gradual decline with time as compared with that of the irrigated plants (Figure 3C). Irrigated plants could hold up to 83.3 ± 1.1% of RWC throughout the experiment, whereas the non-irrigated plants showed a significant decrease, with an RWC of 78.5 ± 5.4% on day 5, 70.1 ± 2.6% on day 10, and 62.6 ± 3.4% on day 15 of stress (Figure 3C). After re-watering, the RWC of the non-irrigated plants were recovered to the levels of the RWC of the irrigated ones. Water withholding significantly reduced the SMC (declined by up to 92.29%) (Figure 3B). However, the decrease in the RWC of the plants appeared less drastic (reduced by 24.4%) (Figure 3C), suggesting a better water holding capacity of *S. hexandrum*.

Decline in the SMC and RWC of plants are the signature phenotype of water deficit (Keyvan, 2010; Soltys-Kalina et al., 2016). However, maintenance of RWC is implicated with water deficit tolerance, as reported on several plant species, such as tomato (Rao et al., 2000), sunflower (Jamaux et al., 1997), and barley (Altinkut et al., 2001). Higher water content is concomitant with osmotic adjustment and elasticity of the cell wall of tissues (Morgan, 1984; Ritchie et al., 1990). Since water deficit is inevitable for *S. hexandrum* in its niche because of seasonal availability of water, maintenance of RWC could be one of the inherent approaches of *S. hexandrum* to cope with stress.

**FIGURE 4:**
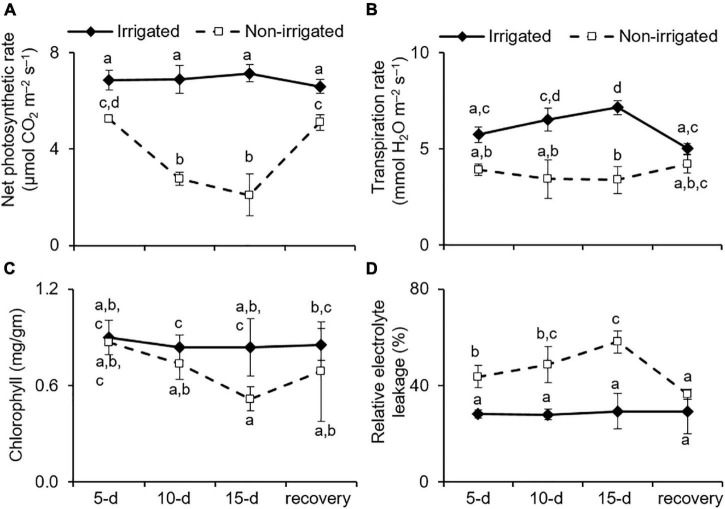
Water deficit negatively impacted the physiology of *S. hexandrum*. **(A)** Net photosynthetic rate. **(B)** Transpiration rate. **(C)** Chlorophyll content. **(D)** Relative electrolyte leakage showing membrane damage. The data values show mean ± SE of three independent biological replicates. Lowercase letters represent statistical significance of differences between the mean values (*P* < 0.05, Duncan’s multiple comparison test).

Under water deficit conditions, plants tend to maintain RWC by preventing transpirational water loss, which is achieved by closing the stomata (Brodribb and Holbrook, 2003). However, this also represses gas exchange, leading to reduced availability of CO_2_ for the Calvin cycle and affects the overall photosynthetic rate (Chaves et al., 2009). As anticipated, the non-irrigated *S. hexandrum* plants exhibited a reduction in transpiration rate, consequently repressing photosynthesis rate upon water deprivation (Figures 4A,B). On day 15 of the water deficit, transpiration rate was reduced by 38.9 ± 17.5%, and the rate of photosynthesis declined by 66.7 ± 16.7% (Figures 4A,B). The rate of transpiration and net photosynthesis was recovered upon re-watering, although they remained significantly lower than that of the irrigated plants (Figures 4A,B).

Given the appearance of lesions, such as wilting and chlorosis of the leaves of water-deprived plants (Figure 3A), the impact of water deficit on chlorophyll content and membrane damage was analyzed. Both chlorophyll content and membrane damage have an impact on photosynthetic efficiency and are implicated in stress tolerance of the plants (Sengupta et al., 2019; Wungrampha et al., 2019; Joshi et al., 2020). The data showed that chlorophyll content was significantly reduced with stress (declined by 32.4 ± 8.7% on day 15 of stress) and recovered by 24.7% after re-watering (Figure 4C). On the contrary, REL, which reflects membrane damage, was significantly higher in the non-irrigated plants as compared to the irrigated plants. The REL showed a dose-dependent increase with stress and could largely recover after re-watering (Figure 4D). The REL of the leaf tissue of irrigated plants did not show any significant change (28.7 ± 0.67%) during the course of the experiment, whereas in the non-irrigated plants, the REL increased to 43.7 ± 4.7 (day 5), 48.7 ± 7.4 (day 10), and 58.1 ± 2.69% (day 15) (Figure 4D). Upon re-watering, the REL of the non-irrigated plants exhibited reduction to 36.47 ± 2.08%, which accounted for 37% recovery (Figure 4D).

*Sinopodophyllum hexandrum* exhibited a typical phenotype of water deficit upon water deprivation, showing reduced photosynthesis and transpiration rate coupled with reduction in chlorophyll content and increased cell death; however, the impact was not that severe. Moreover, the plants could ably recover once they were watered again. The stress tolerance and recovery capability of the *S. hexandrum* species could be due to the maintenance of high relative water content and osmotic adjustments. Such an ability to efficiently recover is a desirable trait for the survival of the species where water deficit is inevitable.

### Increased Proline and Total Soluble Sugar Contents Appear to Protect *S. hexandrum* From Water Deprivation

**FIGURE 5:**
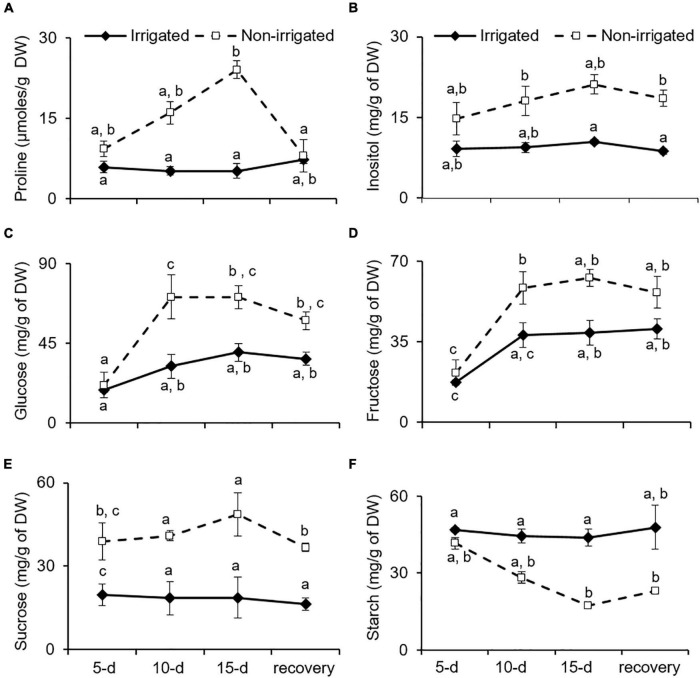
Osmoprotectants tend to accumulate with water deficit in *S. hexandrum*. **(A)** Proline. **(B)** Insolitol. **(C)** Glucose. **(D)** Fructose. **(E)** Sucrose. **(F)** Starch. The data values show mean ± SE of three independent biological replicates. Lowercase letters represent statistical significance of differences between the mean values (*P* < 0.05, Duncan’s multiple comparison test).

Proline is known to be an osmoprotectant, reactive oxygen species scavenger, and molecular chaperone that protects cells from stress-induced damage (Szabados and Savoure, 2010). Plants combat water deficit *via* osmotic adjustment (Morgan, 1984), which is achieved by accumulating secondary metabolites and proline (McCue and Hanson, 1990; Hare and Cress, 1997). Similarly, *S. hexandrum* tended to accumulate proline under water deprivation conditions. In the irrigated plants, proline content was almost constant. However, in the non-irrigated plants, it increased by 24.5 ± 7.9% on day 5, 62.1 ± 8.6% on day 10, and 75.1 ± 2.9% on day 15 of stress, and compared to the irrigated plants, proline content was almost fivefold higher in the leaf tissue on day 15 of stress (Figure 5A). After re-watering for 15 days, proline content declined to 26.3 ± 5.8%, accounting for almost 66% recovery in the leaves of the non-irrigated plants.

Accumulation of sugars also aids in osmotic adjustments and is implicated in recovery from water deficit (Sheffer et al., 1979; Busso et al., 1990; McCue and Hanson, 1990). Given the importance of sugars in stress management, the content of various sugars was analyzed under water deficit conditions in *S. hexandrum* (Figures 5B–E). In the irrigated plants, glucose and fructose exhibited increase with age of the plants, whereas inositol and sucrose remained almost constant. On the contrary, all the four soluble sugars increased with stress in the non-irrigated plants (Figures 5B–E). Upon re-watering, the sugars showed a slight decline in the non-irrigated plants but remained much higher than in the irrigated ones (Figures 5B–E). Inositol levels recovered by 12.2 ± 3.9%, glucose by 18.5 ± 12.4%, fructose by 20 ± 5.9%, and sucrose by 24.4 ± 5.5%. Interestingly, in comparison to the soluble sugars, starch showed a negative trend where it remained almost similar in the irrigated plants, whereas it declined with stress in the non-irrigated plants and exhibited a 2.5-fold decrease on day 15 of stress. Upon re-watering, the starch content in the non-irrigated plants recovered by 24.3 ± 3% but remained almost twofold lesser than in the irrigated plants (Figure 5F).

Increased accumulation of free amino acids, such as proline and soluble sugars, helps in re-adjusting the osmoticum to tackle the water deficit conditions (McCue and Hanson, 1990). Soluble sugars also underscore the capability of plants to recover from water deficit (Sheffer et al., 1979; Busso et al., 1990). Besides acting as osmoprotectants, soluble sugars are also involved in stress signaling during water deficit (Nelson et al., 1998). Accordingly, a higher content of free soluble sugars was observed in water-deprived *S. hexandrum* as compared with the irrigated ones. Higher free sugar content could be either due to increased biosynthesis or increased breakdown of starch (Pattanagul and Madore, 1999; Chaves et al., 2009). In non-irrigated *S. hexandrum*, starch content declined with stress in proportion to the increased levels of free sugars (Figures 5B–F), suggesting a breakdown of starch, as also reported earlier (Pattanagul and Madore, 1999; Chaves et al., 2009). In summary, increased content of proline and soluble sugars in *S. hexandrum* indicates efficient immobilization of amino acids and inclusive use of stored carbohydrate reserve for imparting better survival under water deficit and assistance in recovery afterward.

### Water Deficit Promotes the Accumulation of Podophyllotoxin *via* Transcriptional Upregulation of Genes Involved in Its Biosynthesis and Is Coupled With Changes in Starch and Soluble Sugar Contents

Plants accumulate secondary metabolites when experiencing adverse environmental conditions (Savoi et al., 2016; Liu et al., 2019), which is mainly achieved *via* transcriptional reprogramming of genes involved in the biosynthesis of such metabolites. Therefore, investigating the expression of genes associated with secondary metabolite synthesis contributes to the understanding of stress-responsive mechanisms (Yuan et al., 2012).

**FIGURE 6:**
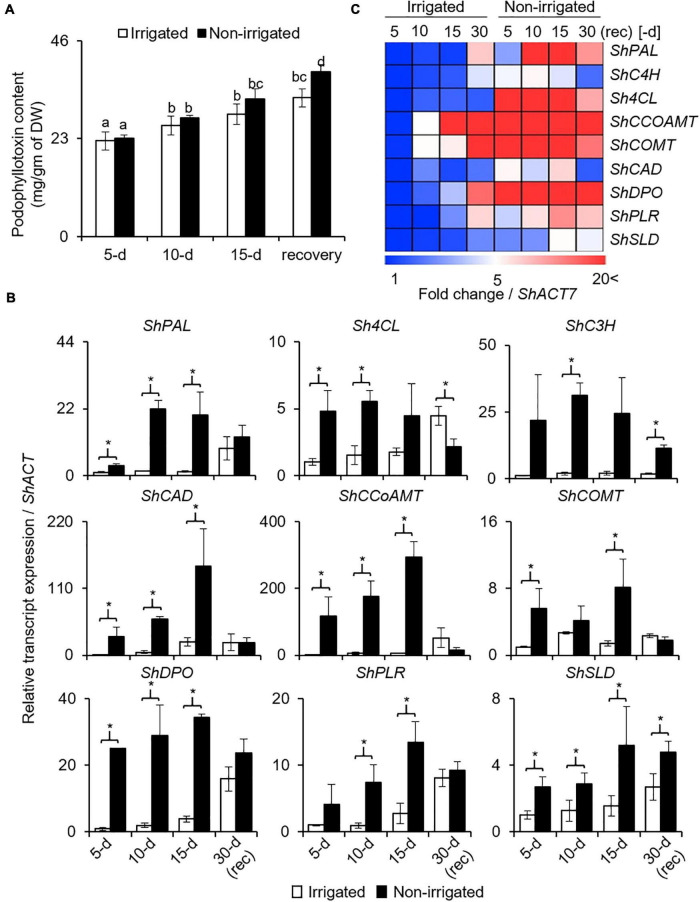
Podophyllotoxin (PTOX) content and expression of biosynthetic pathway genes in *S. hexandrum* increased in response to water deficit. **(A)** PTOX content. Data show mean ± SE of three independent biological replicates. Lowercase letters represent statistical significance of differences between the mean values (*P* < 0.05, Duncan’s multiple comparison test). **(B)** Transcript level expression of PTOX biosynthetic pathway genes. Gene names are prefixed with *Sh* that stands for *Sinopodophyllum hexandrum*. *ShACTIN* was used as an internal control. Data represent mean ± SD of three independent biological replicates. Asterisk show statistical significance of differences between the mean values (*P* < 0.05, Student’s *t*-test). **(C)** Heat map showing the expression of PTOX biosynthetic pathway genes as shown in panel **(B)**.

Podophyllotoxin (PTOX) is a secondary metabolite, and is synthesized *via* phenylpropanoid pathway (Kumari et al., 2014). Given the negative effect of water deficit on growth and physiology, and PTOX being a secondary metabolite, its biosynthesis was anticipated to be affected. In both, irrigated and non-irrigated plants, PTOX content increased with age, irrespective of the watering condition (Figure 6A). In addition, although the difference was not significant on days 5 and 10, 12.1% more accumulation was observed on day 15 of stress in the non-irrigated plants as compared with the irrigated ones (Figure 6A). Upon re-watering, the PTOX content in the non-irrigated plants continued to increase. Interestingly, the rate of increase in the non-irrigated plants was significantly higher with 18.5% more PTOX content than in the irrigated ones (Figure 6A). These data suggested that both water deficit conditions as well as the release, promoted PTOX accumulation in the *S. hexandrum* plants.

Concomitantly, key genes of the phenylpropanoid pathway, namely, *ShPAL, Sh4CL, ShC3H, ShCCoAOMT*, *ShCOMT*, and *ShCAD*, exhibited upregulation upon experiencing water deprivation (Figures 6B,C). Interestingly, all these genes were sensitive to stress and increased rapidly in the initial phase of stress. Thereafter, upregulation was gradual. The increased expression of phenylpropanoid pathway genes indicated the accumulation of secondary metabolites, such as sinapic acid, lignin, and flavanols, which are known to have protective roles (Cheynier et al., 2013; Le Roy et al., 2016; Pereira, 2016). Interestingly, upon re-watering, the genes of the phenylpropanoid pathway exhibited downregulation, although the expression was slightly higher as compared to the irrigated control (Figures 6B,C).

Furthermore, three key genes of the PTOX biosynthetic pathway, *ShDPO*, *ShPLR*, and *ShSLD*, exhibited upregulation in response to water deprivation. Concomitantly, PTOX also accumulated during this time period, suggesting a role of these genes in PTOX biosynthesis and that both genes as well as metabolites responded to the condition of water deprivation (Figure 6A). Similar to phenylpropanoid pathway genes, the expression of PTOX-specific genes was also reduced upon re-watering; however, it remained higher than the irrigated controls. Comparatively, the higher expression of genes of both the phenylpropanoid and PTOX biosynthetic pathways explains why even after recovery, PTOX content was higher in *S. hexandrum* (Figure 6A).

Besides stress conditions, there was an age-dependent gradual increase in the expression of genes involved in both the phenylpropanoid and PTOX biosynthetic pathways. This result indicates why the accumulation of PTOX was increasing even in the absence of stress (Figures 6A–C). Although it needs further investigations, it is likely that increasing hormone levels with age could also play an important role in PTOX accumulation. This assumption agrees with earlier studies where methyl jasmonate-induced ROS generation was shown to stimulate the expression of genes of the PTOX biosynthetic pathway leading to PTOX accumulation (Hazra et al., 2017; Biswas et al., 2021). Changes in the expression level of *ShPLR* and *ShSLD* under drought-like conditions are in accordance with the presence of drought-responsive *cis*-elements in their promoters and indicate their engrossment in stress responses. Experimental validation in the future would narrow down the importance of these individual *cis*-elements.

**FIGURE 7:**
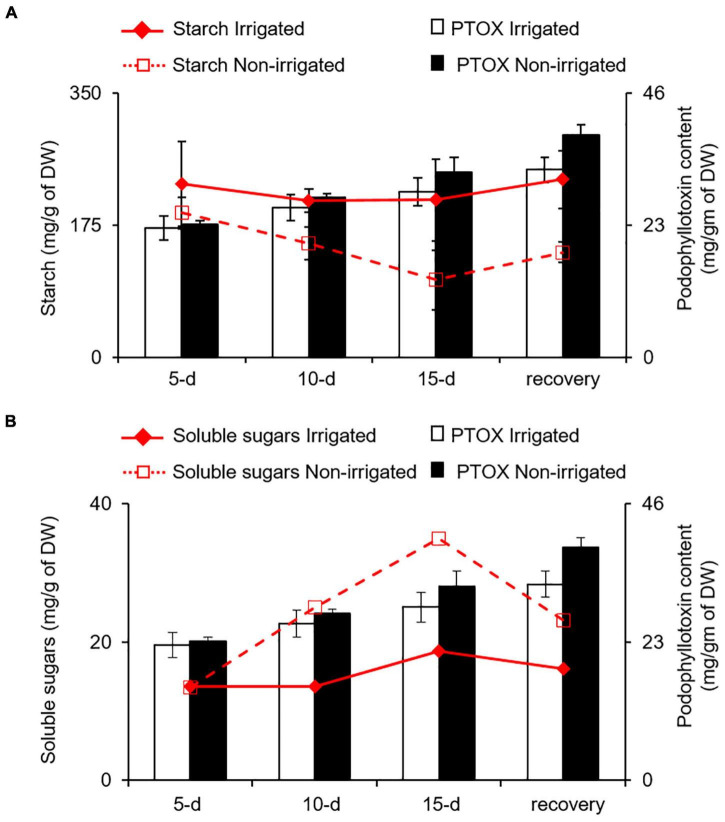
PTOX content in the roots of *S. hexandrum* under water deficit conditions is coupled with starch and soluble sugars contents. Line and bar graph showing the change in PTOX accumulation with **(A)** starch and **(B)** soluble sugar contents. In panel **(B)**, soluble sugars represent the sum of inositol, glucose, and fructose in roots. The values represent the mean ± SE of three biological replicates.

Water deficit is known to influence the expression of genes involved in secondary metabolite biosynthesis leading to differential accumulation of phenolics and other secondary metabolites (Becerra-Moreno et al., 2015; Caser et al., 2019; Babaei et al., 2021; Jogawat et al., 2021). Although water deficit, like other stresses, has a positive impact on secondary metabolite synthesis, the accumulation varies in terms of carbon-based (phenolics and terpenes) and nitrogen-containing (alkaloids and cyanogenic glycosides) secondary metabolites (Al-Gabbiesh et al., 2015; Sanchita et al., 2015). For instance, *Labisia pumila* (Jaafar et al., 2012), *Hypericum resilience* (de Abreu and Mazzafera, 2005), *Trachyspermum ammi* (Azhar et al., 2011), and *Pisum sativum* (Nogués et al., 1998) accumulate total phenolics and flavonoids under water deficit conditions. Similarly, *Salvia officinalis* and *Pinus sylvestris* accumulate monoterpenes (Turtola et al., 2003; Nowak et al., 2010) upon water deficit. On the contrary, increased accumulation of alkaloids in *Senecio longilobus* (Briske and Camp, 1982), *Lupinus angustifolius* (Christiansen et al., 1997), and *Catharanthus roseus* (Jaleel et al., 2007) have been reported under reduced water availability conditions. According to carbon-nutrient balance (CNB) theory, the amount of secondary metabolites in plants is directly regulated by relative abundances of carbon and nitrogen (Bryant et al., 1983; Hamilton et al., 2001). When nitrogen deficiency limits plant growth, carbohydrates tend to accumulate and increase the synthesis and the accumulation of carbon−based secondary metabolites. Conversely, when photosynthesis rate is reduced because of reduced availability of CO_2_ or being under light fluctuation, carbohydrates get a push toward growth, leading to lower carbon-nitrogen (C/N) ratio. This is likely to reduce the synthesis of carbon−based secondary metabolites and increase the synthesis of nitrogenous secondary compounds (Bryant et al., 1983; Herms and Mattson, 1992; Peñuelas and Estiarte, 1998). When *S. hexandrum* is experiencing water deficit, despite having reduced photosynthetic rate, it showed increased expression of phenylpropanoid pathway genes, indicating increased accumulation of carbon-based secondary metabolites, such as PTOX, which seems contrasting to the CNB theory. It is possible that water deficit-induced reduction in SMC might affect nutrient uptake leading to reduced availability of nitrogen and results in higher C/N ratio, favoring carbon-based secondary metabolites. There is a possibility that carbon supply was ensured by increased starch degradation in roots. We assessed this possibility by estimating the starch and soluble sugar content in roots under irrigated and non-irrigated conditions. As observed in the leaves, the roots also exhibited decrease in starch content and increase in soluble sugars (inositol, glucose, and fructose), suggesting increased degradation of starch (Figures 7A,B). Interestingly, accumulation of PTOX was proportional to both decrease in starch and increase in soluble sugar contents with stress (Figures 7A,B). Although it is not clear how increased starch degradation is precisely linked with PTOX accumulation, it has been demonstrated that increased starch degradation compensates for the carbon demand under stress conditions (Pilkington et al., 2015; Jia et al., 2021). Further studies investigating the secondary metabolome in *S. hexandrum* upon stress conditions, such as water deficit, would probably shed light on the pattern of secondary metabolite accumulation in this plant as well as in other high-altitude plants. Nevertheless, it is conceivable that the suppression of growth is liable for the enhanced accumulation of secondary metabolites in the *S. hexandrum* plants exposed to water deficit.

**FIGURE 8:**
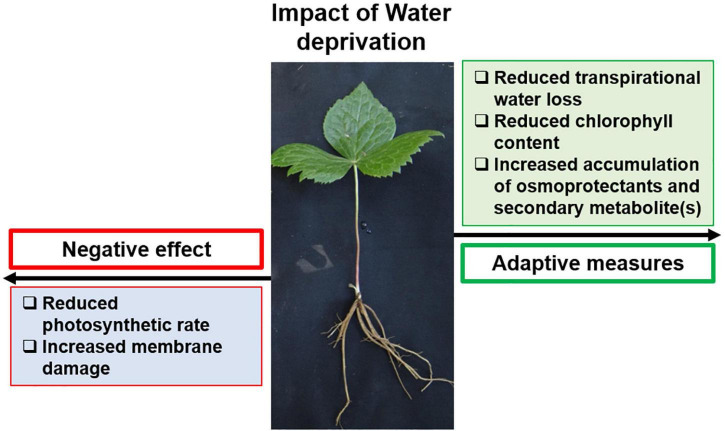
*S. hexandrum* implements an adaptive strategy to sustain under water deficit conditions. Water deprivation reduces soil moisture content and water availability to plants, and induces membrane damage and cell death. To counter this, *S. hexandrum* plants prevent excess transpirational water loss; however, this negatively impacts photosynthetic rate. In response, plants accumulate less chlorophyll, avoiding unnecessary light-harvesting and ROS generation. Besides, osmoprotectants, such as proline and free sugars, accumulate along with the secondary metabolite, PTOX, which helps the plants to sustain under non-permissive conditions.

Summarily, the presence of stress-responsive elements responding to light, temperature, and drought in the promoters of PTOX biosynthesis genes suggested their modulation under respective stress conditions. Accordingly, when subjected to water deficit, *S. hexandrum* showed better adaptation and sustainability (Figure 8). With reduced water availability, *S. hexandrum* plants exhibited reduced transpirational water loss. However, it declined gas exchange and CO_2_ availability that resulted in reduced photosynthetic rate. Reduced water availability also induced membrane damage, leading to cell death. Plants, in turn, accumulated less chlorophyll to avoid unnecessary harvesting of light, which could produce ROS if not used in photochemistry. Besides, osmoprotectants such as proline and free sugars were accumulated along with the PTOX. The increased accumulation of PTOX upon water deficit indicated its possible involvement in stress alleviation. However, this proposition awaits further investigations, which would also illuminate the biological role of PTOX.

## Conclusion

In conclusion, the *in silico* analysis of the upstream regions of two PTOX biosynthesis pathway genes, namely *ShPLR* and *ShSLD*, identified stress-responsive elements implicated in response to water availability, light, and temperature stimuli, indicating that the expression of these genes could be modulated upon these environmental cues. Accordingly, both genes were upregulated under water-deprived conditions. In addition, *S. hexandrum* plants showed better adaptability to reduced water availability. Presence of cognate stress elements in the promoter regions of various genes seems to underscore the molecular basis of the better performance of *S. hexandrum* under water deficit conditions. Detailed characterization of these promoters would provide further insights into their applicability for engineering stress tolerance in other plants. Besides, increased accumulation of PTOX under limited water availability conditions indicates its possible involvement in stress endurance, helping the plant to adapt to non-permissive conditions. However, until now, there has been no direct experimental evidence supporting this proposition. Therefore, it is worth investigating the biosynthesis, steady-state levels, and mobilization of PTOX under stress conditions (namely high light, low temperature, and water deficit) and during recovery.

## Data Availability Statement

The datasets presented in this study can be found in online repositories. The names of the repository/repositories and accession number(s) can be found in the article/Supplementary Material.

## Author Contributions

SK conceived and designed the study and finalized the manuscript. AK performed the experimental work and wrote the initial draft of the manuscript. VD analyzed the data, prepared the figures, and wrote the manuscript. RJ helped in the drafting of the manuscript. All the authors have read and approved the final version of the manuscript.

## Conflict of Interest

The authors declare that the research was conducted in the absence of any commercial or financial relationships that could be construed as a potential conflict of interest.

## Publisher’s Note

All claims expressed in this article are solely those of the authors and do not necessarily represent those of their affiliated organizations, or those of the publisher, the editors and the reviewers. Any product that may be evaluated in this article, or claim that may be made by its manufacturer, is not guaranteed or endorsed by the publisher.

## Footnotes

1
https://www.dna.affrc.go.jp/PLACE/?action=newplace

2
http://bioinformatics.psb.ugent.be/webtools/plantcare/html/

3
http://www.metrohm.com/

## Abbreviations

DW: dry weight
FW: fresh weight
REL: relative electrolyte leakage
RWC: relative water content
SMC: soil moisture content.
