# Supplementary material for: Stress-Responsive cis-Regulatory Elements Underline Podophyllotoxin Biosynthesis and Better Performance of Sinopodophyllum hexandrum Under Water Deficit Conditions
Source: Front Plant Sci. 2022 Jan 4;12:751846. doi: 10.3389/fpls.2021.751846 (PMC8764236; doi:10.3389/fpls.2021.751846)
Supplement: Supplementary file 1 [file Presentation_1.pdf]

Supplementary Figure 1

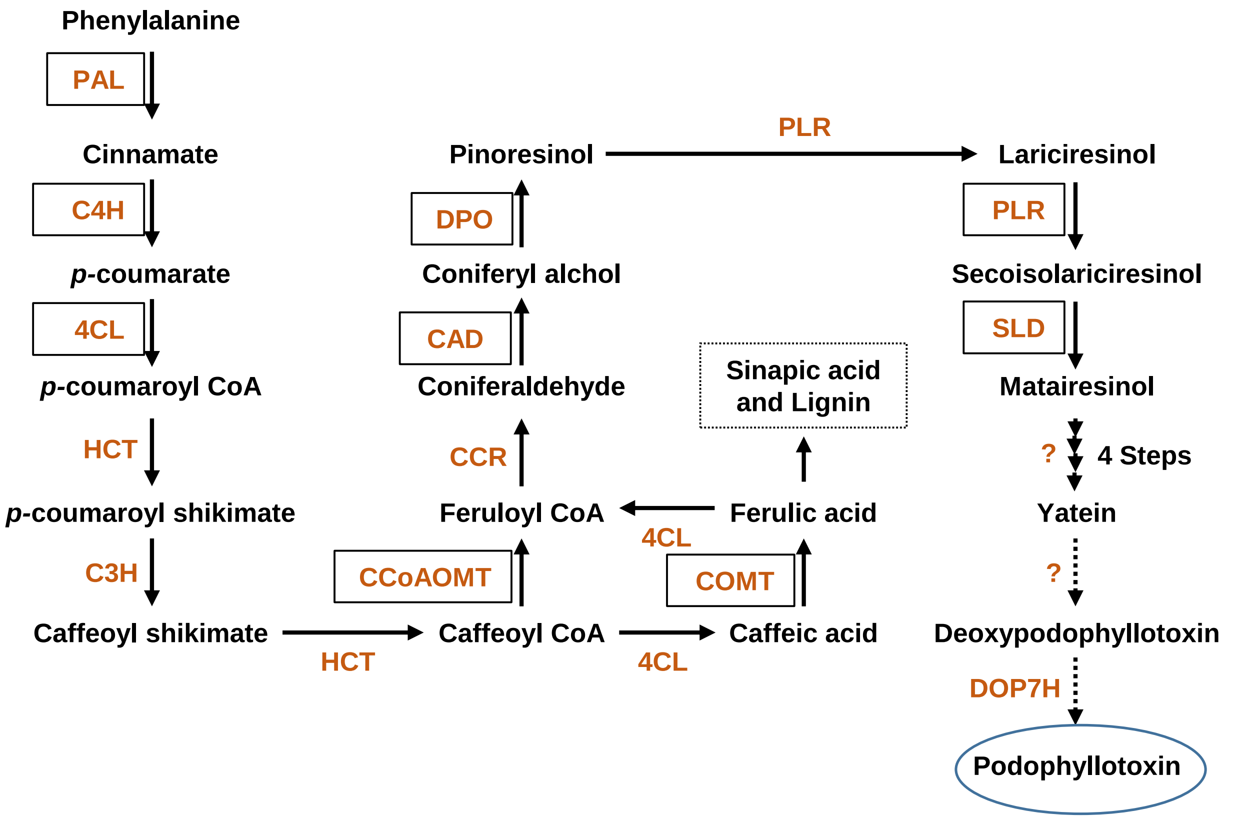

Supplementary Figure 1: Podphyllotoxin biosynthetic pathway. The solid arrows indicate the known steps, whereas the broken arrows indicate putative reactions. The question mark (?) indicates the uncharacterized steps. The expression of the genes presented in black boxes was confirmed during drought. Enzyme abbreviations are as follows; PAL: Phenylalanine ammonia lyase; C4H: cinnamate 4-hydroxylase; 4CL: 4-coumarate: CoA ligase; HCT: p-hydroxycinnamoyl-CoA: quinate shikimate p-hydroxycinnamoyl transferase; C3H: p-coumarate 3-hydroxylase; CCoAOMT: caffeoyl-CoA O-methyltransferase; COMT: caffeic acid 3-O-methyltransferase; CCR: cinnamoyl-CoA reductase; CAD: cinnamyl alcohol dehydrogenase; DPO: dirigent protein oxidase, PLR: pinoresinol–lariciresinol reductase; SLD: secoisolariciresinol dehydrogenase; DOP7H, deoxypodophyllotoxin 7-hydroxylase.

Supplementary Figure 2

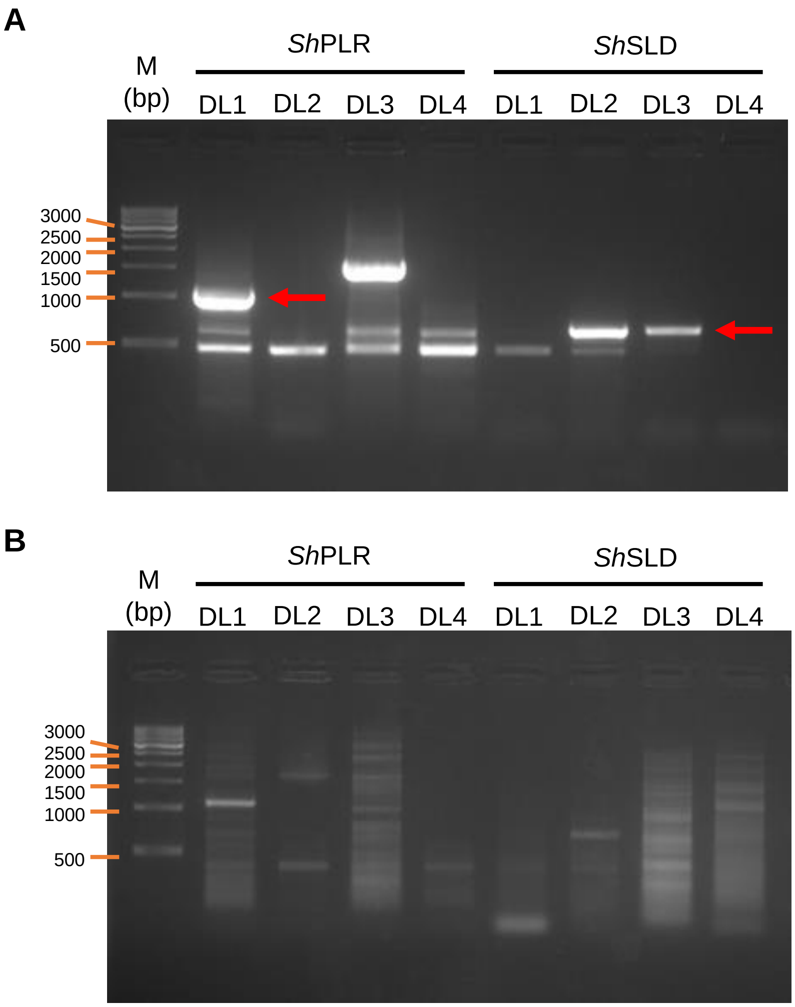

Supplementary Figure 2: Electrophoretic analysis showing amplified products of upstream region of *ShPLR* and *ShSLD*. DNA library 1 (DL1), DL2, DL3, and DL4 represent four Genome Walker libraries prepared from genomic DNA digested with the restriction enzymes; DraI, EcoRV, PvuII and StuI, respectively, for *ShPLR* and *ShSLD*. (A) Primary PCR; (B) Secondary PCR. The cloned fragments are indicated by red arrow. M represents 500 bp DNA size marker.

Supplementary Figure 3

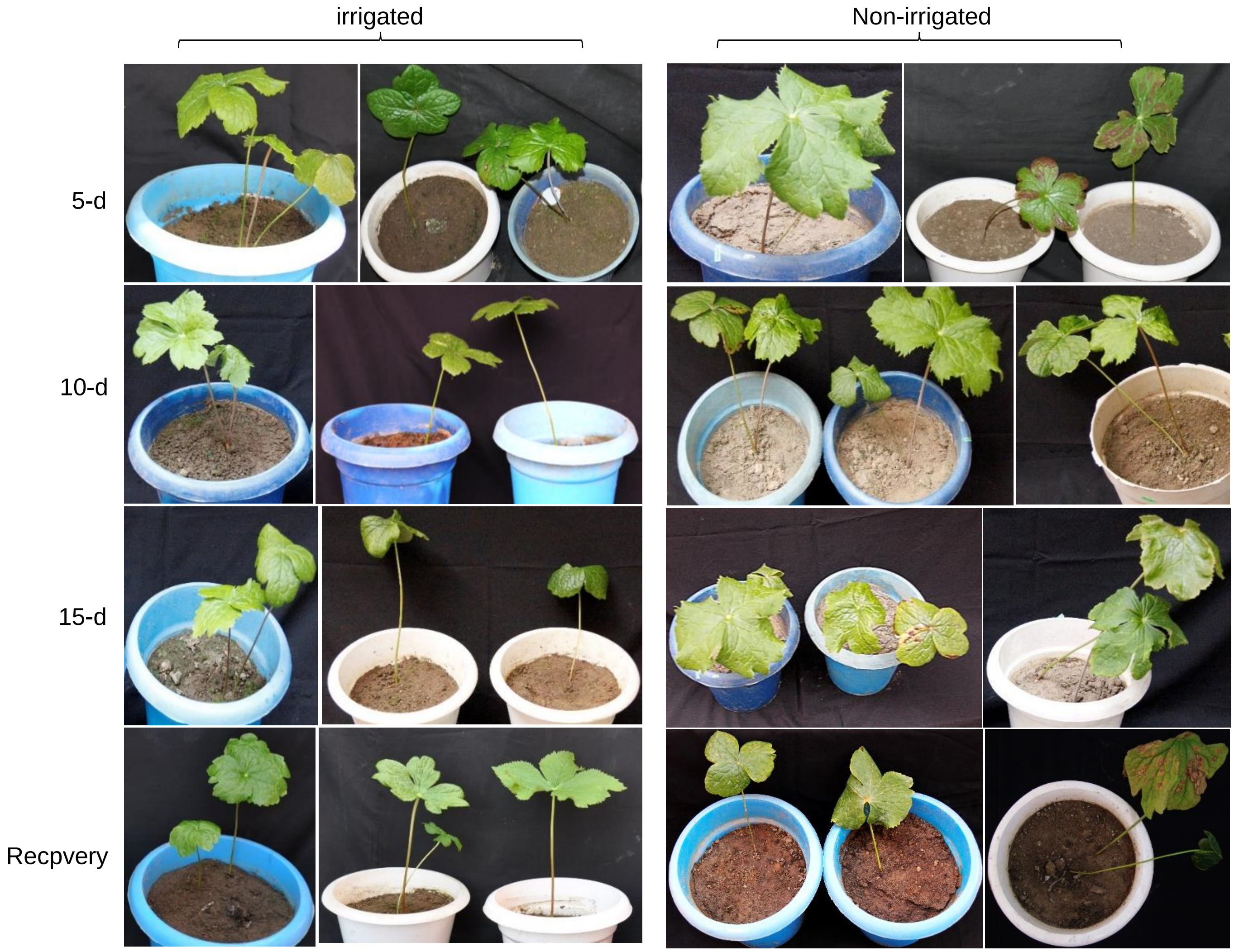

Supplementary Figure 3: Macroscopic phenotype of *S. hexandrum* plants subjected to water deprivation showed a water deficit phenotype. Soil-growing plants, exposed to progressive water deficit by withholding water for 5-, 10-, and 15 days (until the plants showed wilting and chlorosis), were considered as non-irrigated. Plants watered every alternative day were considered as irrigated controls. After 15 days of withholding water, non-irrigated plants were re-watered every alternative day or additional 15 days till the plant exhibited a comparable phenotype as that of irrigated ones. Plants were grown in triplicates and the best representative images are shown in Figure 3A.
