# Supplementary material for: Stress-Responsive cis-Regulatory Elements Underline Podophyllotoxin Biosynthesis and Better Performance of Sinopodophyllum hexandrum Under Water Deficit Conditions
Source: Front Plant Sci. 2022 Jan 4;12:751846. doi: 10.3389/fpls.2021.751846 (PMC8764236; doi:10.3389/fpls.2021.751846)
Supplement: Supplementary file 2 [file Table_1.DOCX]

**Supplementary Table 1.** List of primers used for cloning 5′-upstream regions of *ShPLR* and *ShSLD*

| **Name** | **Sequence** |
| --- | --- |
| *ShPLR* | GSP1: 5′-ATTCTCCTTCCTAGGTACCCTGTACCCC-3′  GSP2: 5′-CCAACAATGAGAACTCTGCTCTTAGCCAT-3′ |
| *ShSLD* | GSP1: 5′- CTTTAGCACCGTAGCGGACGAATAATTT -3′  GSP2: 5′-TGTTGGTAGACGAAGCTGGTGTAGAAGTGG -3′ |
| GW adaptor | 5′-GTAATACGACTCACTATAGGGCACGCGTGGTCGACGGCCCGGGCTGGT-3′  3′-H2N-CCCGACCA-PO4-5′  (supplied by the manufacturer) |
| AP1 Primer | 5′-GTAATACGACTCACTATAGGGC-3  (supplied by the manufacturer) |
| AP2 Primer | 5′-ACTATAGGGCACGCGTGGT-3′  (supplied by the manufacturer) |

GSP: Gene specific primer; GW adaptor: GenomeWalker Adaptor; AP1: Adaptor primer 1; AP2: Nested adaptor primer 2

**Supplementary Table 2.** List of primers used in qRT-PCR analysis.

| **Genes** | **Primer sequences** | **Melting temperature (Tm)** |
| --- | --- | --- |
| *ShActin* | Fwd 5′-ATTACCTGGCCATCAGGAAGA-3′  Rev 5′-GGAAATTGTCCGTGACATGA-3′ | 58°C |
| *ShPAL* | Fwd 5′-CCCTCCATGGTGGCAACTTC-3′  Rev 5′-GGGTTGGGACCACCACTCAA-3′ | 60 °C |
| *Sh4CL* | Fwd 5′-CCGCACCAGAACACAACTGC-3′ Rev 5′-GACGCATGTGCCGGTCGT-3′ | 60 °C |
| *ShC3H* | Fwd 5′-TGGACGAGCAGGGAAGGAG-3′  Rev 5′-CCCTTCGAGTGGCATGTCTG-3′ | 60 °C |
| *ShCOMT* | Fwd 5′-AGCGAAAGCAGGAGAAGGTG-3′ Rev 5′-GCGACGGAGCAGTTTAGGAT-3′ | 59 °C |
| *ShCCoAMT* | Fwd 5′-AGCCGTTTGTGAGAGCCAAT-3′  Rev 5′-AGGCGGAGAGAGGAGTGTTG-3′ | 60 °C |
| *ShCAD* | Fwd 5′-GAAAGCTTGTTATGGTTGGG-3′  Rev 5′-CCATGGCTGTGTTCAAATAA-3′ | 57 °C |
| *ShDPO* | Fwd 5′-ACAGTTGTTGGTGGTACAGG-3′  Rev 5′-TCATCTCAGTTTCCACTCACA-3′ | 59 °C |
| *ShPLR* | Fwd 5′-GAGGCCGTGAAATTGGTTGA-3′ Rev 5′-ACCAAACTCCGATGGCAAGA-3′ | 58 °C |
| *ShSLD* | Fwd 5′-AGGGTTGAGGAATGGCACA-3′  Rev 5′-AGGCCGCTCACATACTTGGA-3′ | 59 °C |
